# Association between perceived workload and mental health among electricians

**DOI:** 10.1192/j.eurpsy.2023.933

**Published:** 2023-07-19

**Authors:** I. Sellami, A. Feki, A. Abbes, M. A. Ghrab, N. Kotti, S. Baklouti, M. L. Masmoudi, K. Jmal Hammami, M. Hajjaji

**Affiliations:** 1 occupational medecine; 2 Rheumatology, Hedi Chaker Hospital, University of Sfax, Sfax, Tunisia

## Abstract

**Introduction:**

The work conditions of electricians have been associated with heavy physical and psychological workloads. It is essential to know the impact of this perceived workload on the mental health of workers.

**Objectives:**

This study aimed to assess the relationship between perceived workload on the mental health of workers.

**Methods:**

The study was conducted among a group of electricians. Data were gathered between January-June 2022 using a self-administered questionnaire including socio-professional characteristics and Kessler Psychological Distress Scale (K6). To assess the perceived workload, we choose the National Aeronautics and Space Administration Task Load Index (NASA-TLX). In this study, we evaluated raw NASA-TLX scores.

**Results:**

Seventy-four workers participated in the study. They were married in 67,6% of cases. The mean age was 39,3 ± 10,5 years. The average job tenure was 15,5 ± 11,2 years. The mean score of K6 was 5,4±4,8. The proportion of respondents with high levels of psychological distress (K6 score of 13 or greater) was 9.5 %. The mean score of mental demand, physical demand, performance, effort, frustration level and temporal demand were respectively 88.8±14, 60.8±23.6, 85.2±13.1, 82.7±15.4, 34.3±29.3 and 61.8±29.2. The frustration level was correlated with high levels of K6 (p = 0.002, r = 0.36).

**Conclusions:**

A high perceived workload was correlated with the altered mental health of workers. Hence, practices and policies should focus on improving working conditions to enhance the mental health of the employees.

**Disclosure of Interest:**

None Declared

